# RdxA Diversity and Mutations Associated with Metronidazole Resistance of *Helicobacter pylori*

**DOI:** 10.1128/spectrum.03903-22

**Published:** 2023-03-21

**Authors:** Yanan Gong, Kangle Zhai, Lu Sun, Lihua He, Hairui Wang, Yahui Guo, Jianzhong Zhang

**Affiliations:** a State Key Laboratory for Infectious Disease Prevention and Control, National Institute for Communicable Disease Control and Prevention, Chinese Center for Disease Control and Prevention, Beijing, China; Yangzhou University

**Keywords:** *Helicobacter pylori*, metronidazole, RdxA, MIC

## Abstract

Metronidazole (MNZ) is administered as first-line antibiotic for Helicobacter pylori eradication therapy; however, increasing resistance to MNZ impaired the efficacy. Increasing the dose of MNZ was recommended to overcome low-level resistance, but it was difficult to determine MNZ resistance level simply based on the *rdxA* gene mutation. In this study, the *rdxA* sequences of 511 clinical H. pylori strains were analyzed to assess the genotypes associated with MNZ resistance. We observed that the prevalences of *rdxA* sequences with missense, nonsense, and frameshift mutations were 70.25, 11.35, and 17.03%, respectively. Regarding the amino acid substitutions, T31E, H53R, D59N, L62V, S88P, G98S/N, R131K, and V172I were present in most strains regardless of the resistance phenotype. The correlation analysis showed R16H/C, Y47C, A67V/T, and V204I substitutions were associated with MNZ resistance. The mutation resulting in RdxA truncation was observed in 36.29% of the resistant strains, and 83.45% of these strains displayed high-level MNZ resistance (MIC > 256 μg/mL). Moreover, all strains with truncated mutation positions before amino acid 70 expressed high-level MNZ resistance. Our results indicated that most amino acid mutations probably contributed to the sequence diversity of RdxA, while R16H/C, Y47C, A67V/T, and V204I were potentially helpful to identify resistant strains. Although it was difficult to determine the mutations associated with MNZ resistance, the prediction of high-level resistance based on truncated characteristics of RdxA might be an important approach, which can effectively avoid H. pylori eradication therapy with unreasonable of MNZ dose increases for patients with high-level drug resistance.

**IMPORTANCE** The increasing resistance to metronidazole impaired the efficacy of Helicobacter pylori eradication, and increasing the dose of metronidazole was recommended to overcome low-level resistance. For patients infected with highly resistant strains, the current empirical treatments, which generally used metronidazole in double doses or more, appeared impossibly to overcome the resistance and would only increase the incidence of adverse effects. Our results indicated that high-level metronidazole resistance was predominant, and almost half of the patients with high-level drug resistance could avoid usage of metronidazole based on the truncated mutations of RdxA sequences, which can effectively avoid H. pylori eradication therapy with unreasonable increases in the metronidazole dose.

## INTRODUCTION

Metronidazole (MNZ) is administered as an inactive prodrug, and its antibacterial activity is based on reduction of the nitro group to form cytotoxic intermediates that damage the DNA, resulting in strand breakage, helix destabilization, unwinding, and ultimately cell death (1, 2). MNZ is used extensively worldwide against Helicobacter pylori infection, which can cause chronic gastritis, peptic ulcer disease, gastric carcinoma, and mucosa-associated lymphoid tissue lymphoma (3, 4).

MNZ is considered the first-line antibiotic for H. pylori eradication therapy; however, increasing resistance to MNZ is a key factor impairing the efficacy of MNZ therapy (5, 6). To improve the eradication rates, the Maastricht IV/Florence Consensus Report recommended that increasing the dose, frequency, and duration of MNZ treatment could partially overcome MNZ resistance, which is predominantly low-level resistance (7). Therefore, it is important to accurately determine the resistance level for MNZ.

Compared to the traditional culture-based test, which is time-consuming and costly, molecular detection allows for rapid analysis directly from biopsy samples. Resistance to MNZ is mainly due to the inactivation of RdxA, an oxygen-insensitive NADPH nitroreductase, which is encoded by *rdxA* gene and can catalyze the reduction of MNZ to form hydroxylamine (8). Thus, an *rdxA*-based molecular approach for prediction of MNZ resistance offers an attractive alternative. However, previous studies reported that *rdxA* mutations did not cluster in a defined region, which limited the use of *rdxA* as a marker for MNZ resistance (9–13).

In order to further identify specific mutations of *rdxA* associated with MNZ resistance, we analyzed the *rdxA* sequences of 511 clinical H. pylori strains isolated from different regions in China.

## RESULTS

### Antibiotic resistance of MNZ.

**FIG 1:**
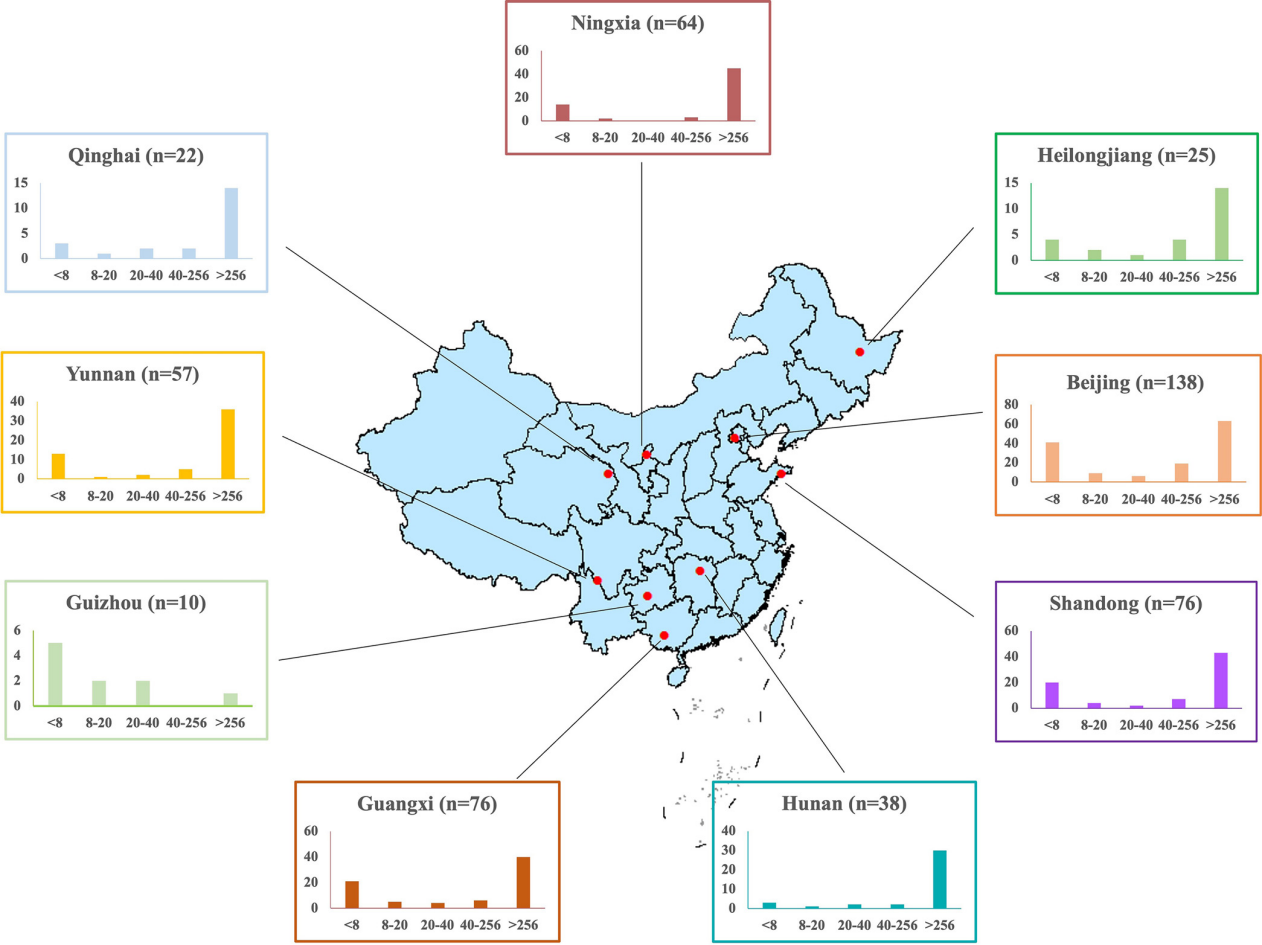
Sample distribution and metronidazole resistance of H. pylori from different regions of China. *n*, the number of strains. The numbers on the horizontal axis indicate the MIC of MNZ. The vertical axis indicates the numbers of strains. The regions indicated the different provinces of China. The five strains from other regions are not mentioned on the map.

The overall resistance rate was 74.95% (383/511) for MNZ, and the resistance rates in different geographic regions are summarized in Table 1. The highest resistance rate, which was up to 92.11%, was observed in Hunan province (Fig. 1).

**TABLE 1:** Resistance rates of MNZ in different regions from China

Region	No. of strains	Rate (%)^*a*^	
		Resistance (*n*)	Susceptible (*n*)
Beijing	138	70.29 (97)	29.71 (41)
Guangxi	76	72.37 (55)	27.63 (21)
Heilongjiang	25	84 (21)	16 (4)
Hunan	38	92.11 (35)	7.89 (3)
Qinghai	22	86.36 (19)	13.64 (3)
Ningxia	64	78.13 (50)	21.87 (14)
Shandong	76	73.68 (56)	26.32 (20)
Yunnan	57	78.95 (45)	21.05 (12)
Guizhou	10	50 (5)	50 (5)
Other regions	5	0	100 (5)
Total	511	74.95 (383)	25.05 (128)

a *n*, the number of strains.

**FIG 2:**
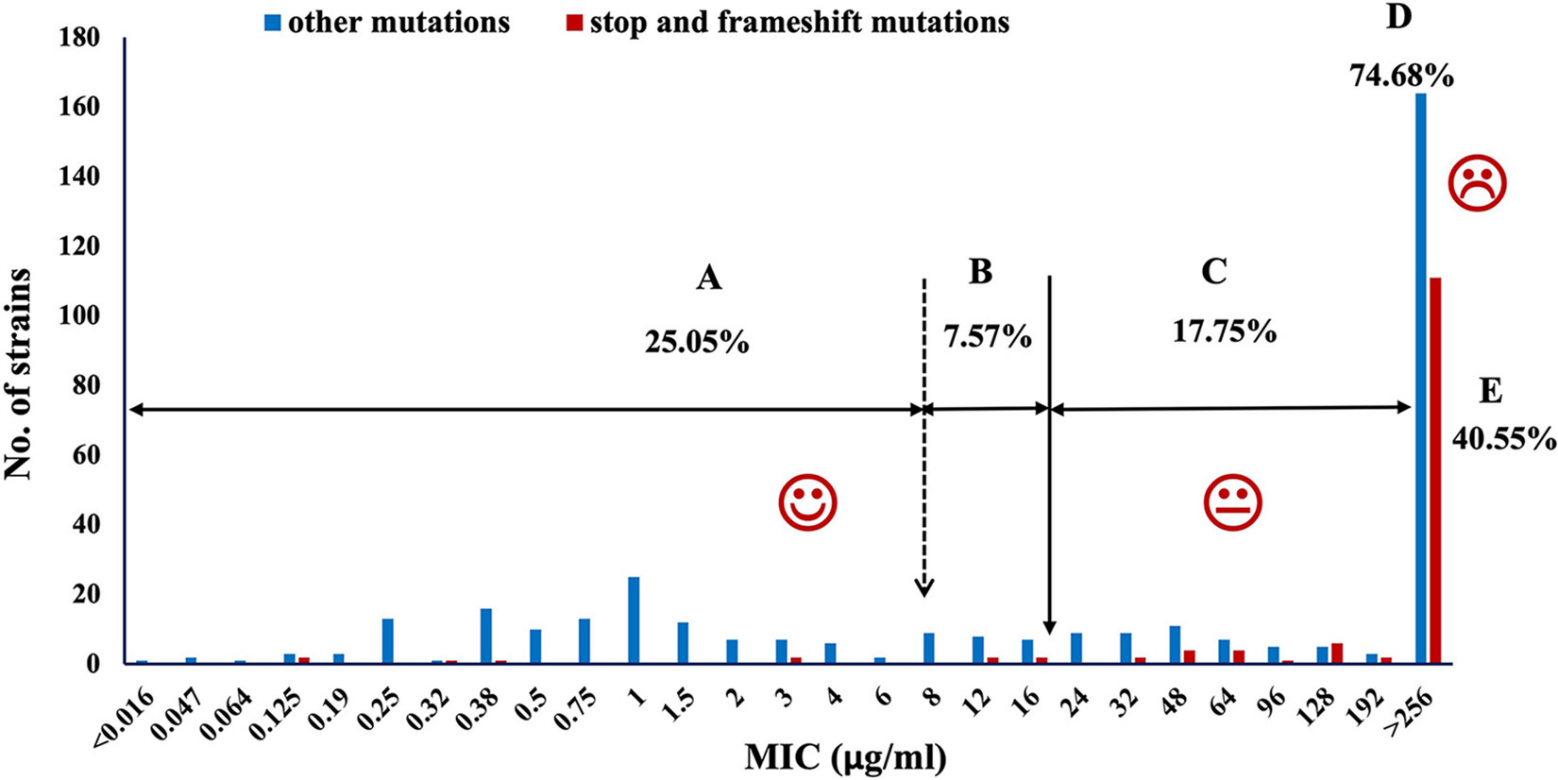
Distribution of MICs of all 511 strains. The dotted line indicates the breakpoint of MNZ resistance. The solid line indicates an MIC of 20 μg/mL, which was the peak plasma concentration of traditional triple therapy. The red bars indicate strains with stop and frameshift mutations, while the blue bars indicate strains with amino acid substitutions.

Regarding the distribution of MIC values, the strains with MICs below 8 μg/mL accounted for 25.05% (labeled A in Fig. 2), which were considered susceptible for MNZ. The strains with MICs ranging from 8 to 20 μg/mL and 20 to 256 μg/mL accounted for 7.57% (29/383) (labeled B in Fig. 2) and 17.75% (68/383) (labeled C in Fig. 2), respectively. In addition, there were 286 (74.68%) strains with the MICs above 256 μg/mL (labeled D in Fig. 2).

Except for Guizhou province with a small number of strains, the distribution patterns of MIC values in the different regions were similar, with predominant high levels of MNZ resistance (MIC > 256 μg/mL) (Fig. 1).

### Mutations and diversity of RdxA sequences.

Compared to the RdxA sequence in strain 26695, all strains tested possessed mutated RdxA sequences regardless of resistance pattern (see Table S1 and S2 in the supplemental material). Missense, nonsense, and frameshift mutations were observed in 359 (70.25%), 58 (11.35%), and 87 (17.03%) strains, respectively. In addition, the remaining 7 (1.37%) strains had several amino acids deletion.

**FIG 3:**
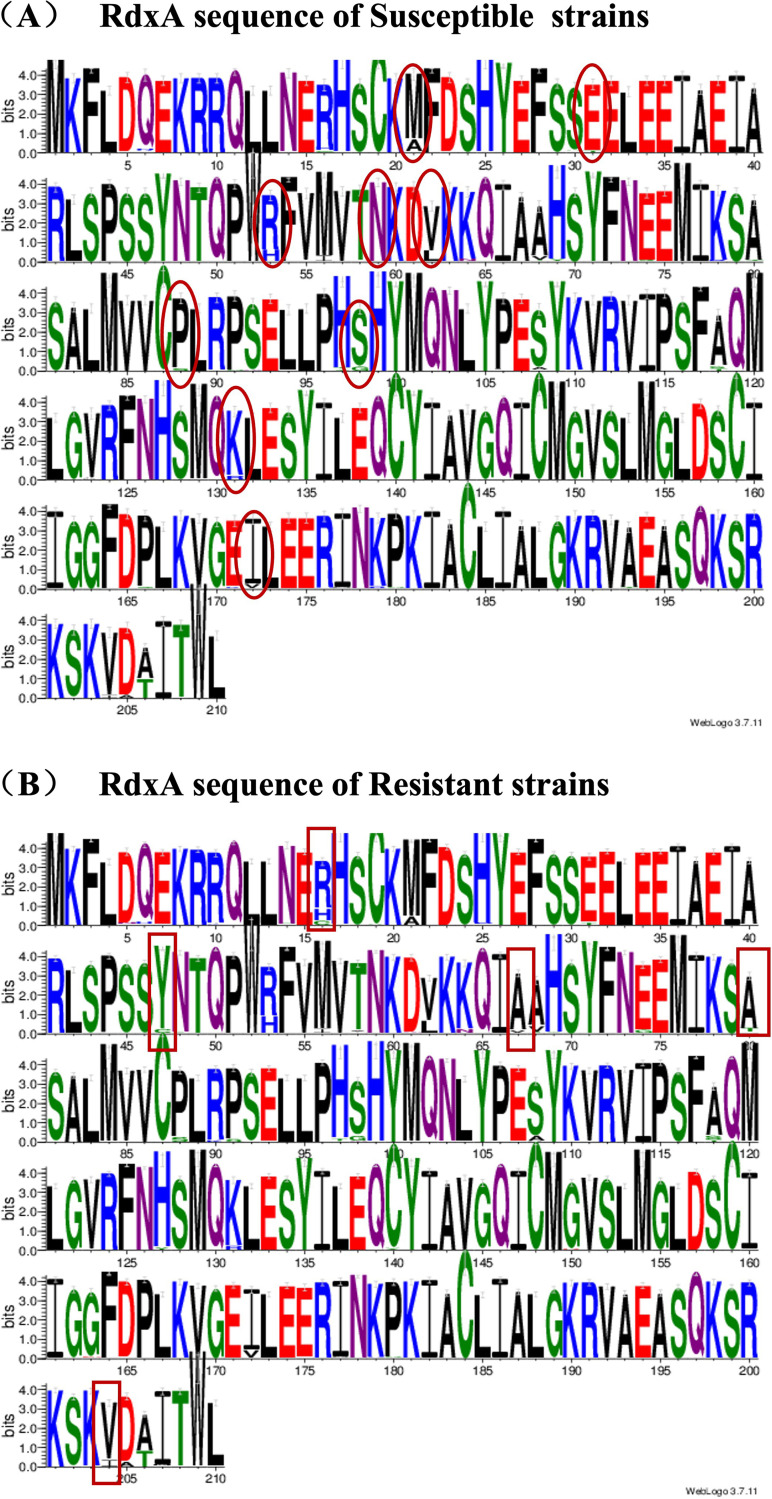
Polymorphism analysis of RdxA sequences in susceptible and resistant strains. (A) RdxA sequences of susceptible strains. (B) RdxA sequences of resistant strains. The figure was drawn by using WebLogo 3. The horizontal axis indicates the amino acid positions. The different amino acids on the same position show the polymorphism of sequences. The amino acids in the circles displayed in panel A were observed in both susceptible and resistant strains, which might contribute to sequence diversity, while the amino acids in boxes displayed in panel B were significantly prevalent in resistant strains, which were relevant to MNZ resistance.

We further performed diversity analysis on RdxA sequences of 359 strains with missense mutations, and the results showed that there were obvious differences for several amino acids from the sequences of RdxA in susceptible or resistant strains, such as amino acids R16, M21, R53, A68, and I172 (Fig. 3).

According to the phylogenetic analysis, the Chinese and European strains were obviously distinguished based on RdxA sequence regardless of the resistance phenotype. Moreover, the strains were not clustered together based on geographic region in China, except for some strains from Ningxia province, indicating that significant differences of RdxA sequences existed even in the same region (see Fig. S1).

### Mutations of RdxA and MNZ resistance.

In our study, 359 strains carried intact RdxA sequence with amino acid substitutions, including 237 MNZ-resistant strains and 122 MNZ-susceptible strains. The alignment of RdxA sequences from resistant strains with those from susceptible strains showed that several amino acid changes, including T31E, H53R, D59N, L62V, S88P, G98S/N, R131K, and V172I, were present in most strains regardless of the resistance pattern (Table 2). The D59N substitution was observed in all strains examined; therefore, this mutation was not implicated in MNZ resistance.

**TABLE 2:** Distribution of amino acid changes and correlation analysis between amino acid mutation and MNZ resistance in our strains^*a*^

Amino acid change	No. (%) of strains		*P*	OR	95% CI
	Resistant (*n* = 237)	Susceptible (*n* = 122)			
R16H/C	63 (26.58)	10 (8.2)	**<0.05**	4.055	1.977–8.233
M21A/I	24 (10.13)	22 (18.03)	<0.05		
T31E	226 (95.36)	118 (96.72)	>0.05		
Y47C	12 (5.06)	1 (0.8)	**<0.05**	6.453	0.829–50.226
H53R	175 (73.84)	100 (81.97)	>0.05		
D59N	237 (100)	122 (100)	>0.05		
L62V	181 (76.37)	98 (80.33)	>0.05		
A67V/T	14 (5.91)	0	**<0.05**	8.206	1.071–62.874
A80T/S	22 (9.28)	3 (2.45)	**<0.05**	4.059	1.19–13.843
S88P	217 (91.56)	116 (95.08)	>0.05		
G98S/N	164 (69.2)	113 (91.87)	<0.05		
R131K	221 (93.25)	114 (92.68)	>0.05		
V172I	208 (87.76)	115 (93.5)	>0.05		
V204I	27 (11.39)	5 (4.07)	**<0.05**	3.009	1.128–8.021

a *n*, number of strains. Differences with a probability (*P*) value of <0.05 were considered statistically significant (indicated in boldface). “R16H/C” in the amino acid change column indicates that the arginine at position 16 was changed to histidine or cysteine. The correlation analysis showed the association between each amino acid mutation and MNZ resistance using SPSS. OR, odds ratio; CI, confidence interval.

The R16H/C, Y47C, A80T/S, or V204I substitution occurred in both susceptible and resistant strains in our study, but with a higher prevalence in resistant strains (*P* < 0.05). In addition, A67V/T mutation (5.91%) was present only in resistant strains. In contrast, M21A/I or G98S/N substitution was significantly prevalent in susceptible strains (*P* < 0.05) (Table 2). Furthermore, correlation analysis showed that amino acid mutations, including R16H/C, Y47C, A67V/T, A80T/S, and V204I, exhibited a significant association with MNZ resistance, especially A67V/T substitution (odds ratio [OR] 8.206; 95% confidence interval [95% CI] = 1.071 to 62.874, *P* < 0.05) (Table 2). The MCMCglmm model suggested that A67V/T had the largest effect on MNZ resistance (post.mean, −903.6216), followed by Y47C, R16H/C, A80T/S, and V204I (Table 3).

**TABLE 3:** Analysis of amino acid mutation effect on MNZ resistance^*a*^

Mutation	post.mean value	95% CI		pMCMC
		Lower	Upper	
R16H/C	−10.1835	−35.3142	−0.2683	<0.001
Y47C	−17.0241	−63.1198	0.5760	0.0104
A67V/T	−903.6216	−2,296.2811	−3.2995	<0.001
A80T/S	−9.2459	−37.2789	1.3897	0.0545
V204I	−8.8217	−31.6549	1.4343	0.0301

a The effects of different mutations on MNZ resistance are indicated by the post.mean value. The smallest value has the largest effect on MNZ resistance. CI, confidence interval; pMCMC, *P* (MCMC) value.

Of the 237 resistant strains, the strains with MICs ranging from 8 to 20 μg/mL and 20 to 256 μg/mL accounted for 10.13% (24/237) and 20.68% (49/237), respectively. Moreover, there were 164 (69.2%) strains with an MIC above 256 μg/mL, and only the prevalence of the M21A/I substitution was significantly different between high-level and low-level MNZ-resistant strains (*P* < 0.05).

In present study, the mutations resulting in RdxA truncation were observed in 6 susceptible strains, as well as 36.29% (139/383) of the resistant strains, including 57 strains with nonsense mutations and 82 strains with frameshift mutations. The difference between susceptible and resistant strains was statistically significant (*P* < 0.05). Among the 139 resistant strains, there were 97 strains (69.78%) with the mutation sites before amino acid 70. The RdxA reading frame was interrupted by stop codon at different positions, predominantly including positions at 50 (23 strains), 103 (4 strains), and 146 (7 strains). Moreover, 85.96% of the nonsense mutations were amino acid Q to stop codon change.

Combined with the MIC, the strains with MICs ranging from 8 to 20 μg/mL and 20 to 256 μg/mL accounted for 2.88% (4/139) and 13.67% (19/139), respectively. Moreover, 116 (83.45%) strains displayed high-level MNZ resistance (MIC > 256 μg/mL) and accounted for 30.28% (116/383) of all resistant strains. RdxA truncation was dominantly more prevalent in MNZ-resistant strains with high MICs than in those with low MICs (*P* < 0.05). In addition, 40.55% (116/286) of the high-level MNZ resistance was caused by RdxA truncation (labeled E in Fig. 2). Regarding on the mutation position, we found all strains with truncated mutation positions before amino acid 70 showed high-level MNZ resistance (MIC > 256 μg/mL), which accounted for 83.62% (97/116) of the strains with MIC above 256 μg/mL.

## DISCUSSION

The current quadruple therapy for H. pylori infection has became widely used throughout the world; however, the success rate has fallen below 80% in recent years (14, 15). The emerging rates of antimicrobial resistance become a significant challenge for the management of H. pylori infection. The global resistance rate of MNZ ranged from 10 to >90%, depending on geographic regions and patient groups with various MNZ use patterns (12, 16–18). In the present study, the resistance rate of MNZ was 74.95% and even up to 92.11% in Hunan province, which may be attributable to the frequent use of MNZ in China.

Despite its high resistance, MNZ has remained a reliable drug for the treatment of H. pylori because of its medicinal property and low cost (1). Traditional triple therapy recommended a 7-day, twice-daily course of a dose with MNZ at 400 mg or 500 mg for H. pylori eradication, leading to the peak plasma concentration reaching 20 μg/mL (19, 20). When MNZ is administered in a maximum safe dose of 2 g/day either in a large single dose or in smaller repeated doses, the peak serum level could reach 40 μg/mL (19, 21). Currently, a 500-mg MNZ dose regimen administered three or four times daily, with extended duration to 10 to 14 days, is recommended, and this treatment probably yields eradication rates ranging from 85 to 94% (13, 22, 23). In this study, there were 7.57% of resistant strains, as well as all susceptible strains, with an MIC of <20 μg/mL, which could be successfully eradicated with standard triple therapy (labeled A and B in Fig. 2). Our study revealed that for patients infected with susceptible and low-level-resistance strains, a dose of 800 to 1,000 mg/day might be a reasonable option, as well as causing few side effects. We observed there were 17.75% resistant strains with MICs ranging from >20 to <256 μg/mL, which could probably be eradicated by increasing the concentration of MNZ as recommended (labeled C in Fig. 2). However, the eradication rates might be influenced by increasing resistance level for MNZ, and simultaneously the adverse effects increased, which would reduce patient compliance, as well as eradication efficacy. Therefore, the benefits from current therapy with increasing MNZ concentration were less predictable.

In addition, resistant strains with a high MIC (>256 μg/mL) accounted for 74.68% (labeled D in Fig. 2), which should avoid the use of MNZ, and current empirical treatments, which generally used MNZ in a double dose or more, appeared to overcome the resistance. For patients infected with highly resistant strains, it seems reasonable to consider other antimicrobial agents, and current regimens would only increase the incidence of adverse effects, especially cerebropathy (24, 25). Thus, the choice of regimens should depend on the MIC of MNZ.

It is accepted that MNZ resistance is predominantly caused by mutations in RdxA (8). Here, 70.25% of our strains contained amino acid substitutions of RdxA, and more than half of the amino acid positions could produce changes. We found that the majority of the mutations occurred infrequently, while several amino acid substitutions, such as T31E, D59N, L62V, and R131K, were present in most of our strains. Previous study reported that the natural genetic diversity of H. pylori *rdxA*, which was about 5 to 8%, could also contribute to the amino acid changes. There is considerable genetic *rdxA* sequence diversity between different MNZ strains (10, 26–28). Moreover, previous study using allelic replacement of *rdxA* showed that amino acid substitutions (M21A, L62V, G98S, and A206T) failed to transfer the MNZ-resistant phenotype, suggesting that these substitutions are not important for MNZ resistance (11). Thus, we conclude that most amino acid mutations might contribute to genetic diversity other than MNZ resistance. The phylogenetic findings of RdxA also confirmed a high genetic diversity, as well as the phylogenetic evolution, which indicated that these amino acid changes might also be phylogenetically related, consistent with previous studies (11, 29).

To identify the mutations associated with MNZ resistance, the RdxA sequences of susceptible and resistant strains from China were analyzed. R16H/C, Y47C, A67V/T, A80T/S, or V204I substitution was more prevalent in resistant strains, and correlation analysis indicated a significant association between these mutations with MNZ resistance, especially the A67V/T substitution with the largest effect on MNZ resistance. Previous studies showed that R16H mutation was involved in a reduction of cofactor flavin mononucleotide (FMN) affinity and the formation of the dimer formation, thus potentially contributing to MNZ resistance (30, 31), while Y47C was expected to be associated with redox function of the enzyme and cause protein destabilization (30). Moreover, V204I substitution was first reported to probably be associated with MNZ resistance in our study. Importantly and consistent with previous studies which reported a A67V/T change was observed only in resistant strains (8, 27, 32), our study further confirms the association between this mutation and MNZ resistance, based on the large number of strains. Interestingly, the M21A/I change, which appeared to be associated with MNZ resistance in other studies (26, 33), was more prevalent in our susceptible strains.

Besides the amino acid substitution, RdxA truncation caused by nonsense and frameshift mutations was commonly associated with MNZ resistance (11, 34–37). In our study, 36.29% of the resistant strains harboring nonsense and frameshift mutations might yield truncated RdxA, as well as most strains exhibiting high-level MNZ resistance (MIC > 256 μg/mL), and accounted for 40.55% of all strains with high-level resistance (labeled E in Fig. 2). Our results indicated that almost half of the predominant patients with high-level drug resistance could avoid usage of MNZ based on the truncated mutations of RdxA sequences.

Moreover, we found that all strains with truncated mutation positions before amino acid 70 were high MNZ resistance (MIC > 256 μg/mL). The structure of RdxA reveals that the core functional homodimer is formed by a five-stranded sheet, and the peptide with a length of 70 amino acids contains only β1 sheet. In addition, the amino acids R16, S18, N73, I160, K198, G162, and R200 are critical for the interaction between RdxA and FMN, which are necessary for its metabolic activity (30). Truncated peptides of <70 amino acids potentially contribute to the reduction of cofactor FMN affinity and dimer stability. We speculated that the position of truncation potentially contributed to the various levels of MNZ resistance, and the truncated peptides of <70 amino acids completely lost the RdxA function and finally degraded rapidly.

In conclusion, based on an *rdxA* sequence analysis of 511 strains, our study assessed the genotype associated with MNZ resistance, phylogenetic evolution, and epidemiological study. Most amino acid mutations, such as T31E, L62V, and R131K, contributed to the genetic diversity of RdxA, while R16H/C, Y47C, A67V/T, and V204I substitutions were associated with MNZ resistance. These mutations were potentially helpful for identifying resistant strains. Further study with more clinical strains, as well as allelic replacement of *rdxA*, will be necessary to confirm the mutations reported here and to investigate the mechanisms of these mutations associated with MNZ resistance, such as how they affect the stability and function of RdxA protein. Also, V204I substitutions may potentially be used to identify MNZ resistance in H. pylori.

The association of MNZ resistance with mutations of *rdxA* is still a controversial topic, and it is a challenge to establish molecular diagnostic approaches to determine MNZ based on the *rdxA* gene. In addition, our study suggests that the administration of MNZ with increased doses might fail to improve the therapy efficacy in patients with high-level MNZ resistance.

Tailored therapy that determines the MIC in advance is encouraged, and if standard culture and susceptibility testing is not possible, determination of the MIC based on the truncated characteristics of RdxA should be an important approach, since more than one-third of the MNZ-resistant strains could be identified simply based on *rdxA* gene sequencing. The best advantage of this method is that all drug-resistant strains detected are highly resistant. In fact, in this case, RdxA protein has lost its metabolic activity to MNZ; thus, it can effectively avoid H. pylori eradication therapy with an unreasonable increase in MNZ dose for such patients.

## MATERIALS AND METHODS

### Strains.

A total of 511 H. pylori clinical strains, including 138 from Beijing city, 76 from Shandong province, 76 from Guangxi province, 64 from Ningxia province, 57 from Yunnan province, 38 from Hunan province, 25 from Heilongjiang province, 22 from Qinghai province, 10 from Guizhou province, and 5 from other regions (Fig. 1), were selected from the National H. pylori Strain Bank of China. These strains were isolated from the gastric biopsy specimens of patients with gastrointestinal diseases who underwent upper gastrointestinal endoscopy from 2013 to 2019. These geographical regions could cover most provinces of China and, based on the defined regions, strains with clear background information were selected from different regions.

The study was approved by the Ethics Committee of National Institute for Communicable Disease Control and Prevention, Chinese Center for Disease Control and Prevention. Written informed consent was obtained from all patients.

### MNZ susceptibility test.

The MNZ MIC was determined by the Etest method. After 48 h of growth, isolates were suspended in sterile saline, and the McFarland turbidity was adjusted to 2.0. The bacterial suspension was spread onto Karmali blood agar plates (with 5% fresh defibrinated sheep blood), and MNZ Etest strips were added, which were then incubated for 48 h under microaerobic conditions. Based on EUCAST guidelines, strains with an MIC of >8 μg/mL were considered resistant to MNZ.

### Extraction of genomic DNA and *rdxA* sequencing.

The genomic DNA was extracted using QIAamp DNA minikit (Qiagen, Germany) according to the manufacturer’s instructions. The PCR was carried out in a volume of 25 μL, and amplification was performed as follows: 94°C for 5 min; followed by 35 cycles of 94°C for 45 s, 54°C for 45 s, and 72°C for 45 s; and, finally, 72°C for 5 min. The primer set was F-GCAGGAGCATCAGATAGTTCT and R-GGGATTTTATTGTATGCTACAA, which would yield a product size of 886 bp, including the *rdxA* gene of 630 bp. The PCR products were sequenced by the Sangon Company (Shanghai, China) and translated to amino acid sequences using Primer 5.0 software. Multiple sequence alignment with HP0954 from strain 26695 was performed by using alignment software.

### Sequence diversity analysis on RdxA.

The diversity of RdxA sequences was analyzed using the program WebLogo 3.

Mega software (https://www.biomart.cn/experiment/430/586/589/2714648.htm) was used to create a phylogenetic tree of RdxA sequences from Chinese and foreign strains. Management and visualization were performed using itol (https://itol.embl.de/), with the following options: branch and label colors, size, legend, etc. The accession numbers for the RdxA sequences of 86 isolates from Indonesia, Germany, Argentina, and Switzerland are LC174777 to LC174814, AJ305346 to AJ305358, DQ062162 to DQ062182, and AF180395 to AF180424.

### Statistical analysis.

The mutation frequency of amino acid was quantified by using the chi-square test. Differences with a probability (*P*) of <0.05 were considered statistically significant. Correlation analysis between amino acid mutations and MNZ resistance was performed using SPSS 25.0 software. We used the Bayesian mixed-model R-package MCMCglmm (v2.32; Hadfield 2010) to investigate the effects of these mutations on MNZ resistance. We fitted the mutations as fixed effects and the strains as a random effect. The prior residual variance was fixed at 1 as “R = list (V = 1, fix = 1)” and a weak prior for random effect was used as “G1 = list (V = 1, nu = 1, alpha.mu = 0, alpha.V = 100)”.
