# Supplementary material for: RdxA Diversity and Mutations Associated with Metronidazole Resistance of Helicobacter pylori
Source: Microbiol Spectr. 2023 Mar 21;11(2):e03903-22. doi: 10.1128/spectrum.03903-22 (PMC10100817; doi:10.1128/spectrum.03903-22)

Figure S1. The phylogenetic analysis on Chinese strains and foreign strains.

(A) phylogenetic analysis on susceptible strains; (B) phylogenetic analysis on resistant strains. The different colors in the phylogenetic trees indicated different geographic regions. The strains in the circle of (A) indicated those from foreign countries. The red circle in (B) indicated the strains from Ningxia province in China.

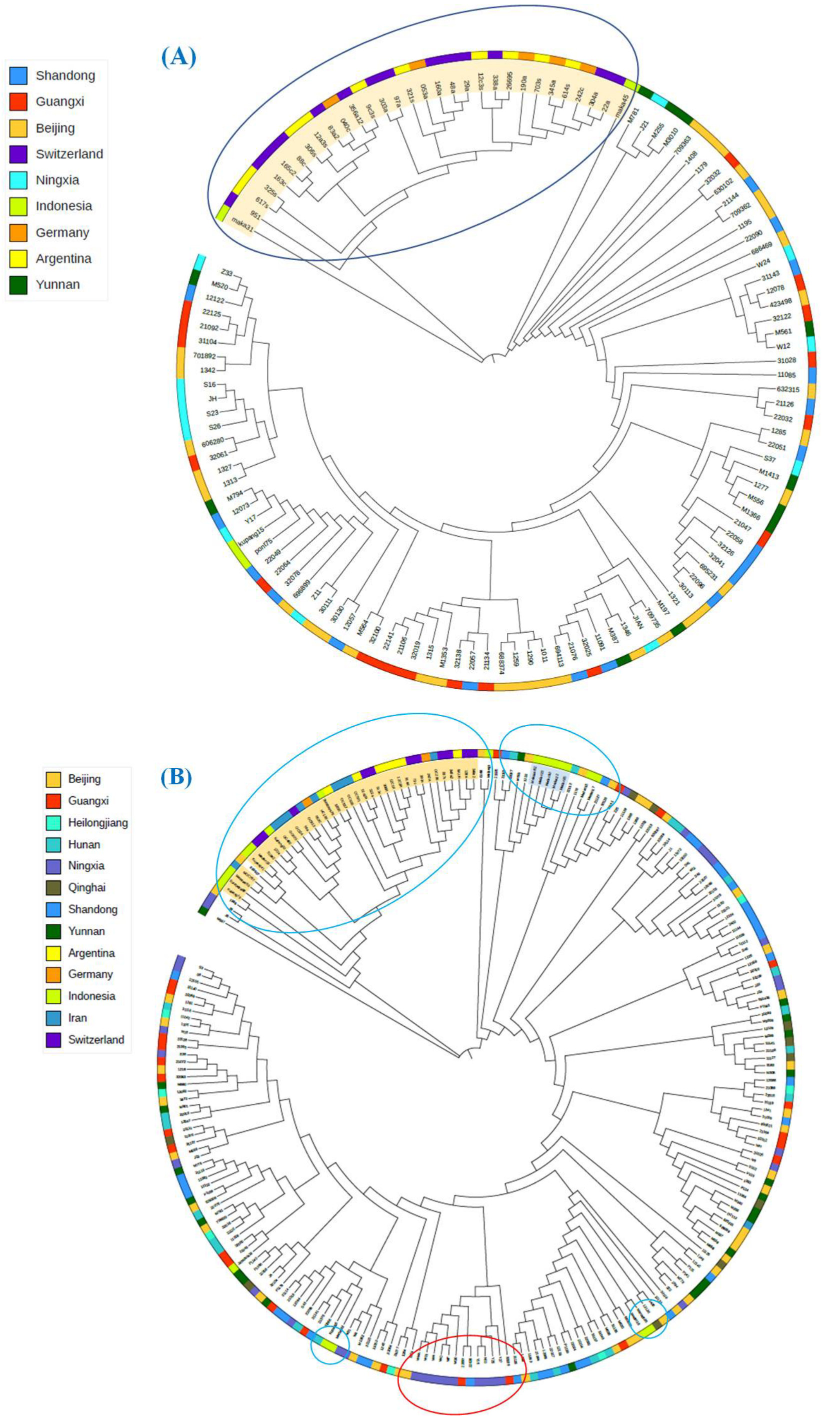

Supplement: Supplemental file 1 — Fig. S1. Download spectrum.03903-22-s0001.pdf, PDF file, 1.5 MB [file spectrum.03903-22-s0001.pdf]
